# Chitosan and nematophagous fungi for sustainable management of nematode pests

**DOI:** 10.3389/ffunb.2022.980341

**Published:** 2022-10-24

**Authors:** Raquel Lopez-Nuñez, Marta Suarez-Fernandez, Federico Lopez-Moya, Luis Vicente Lopez-Llorca

**Affiliations:** ^1^ Department of Marine Sciences and Applied Biology, Laboratory of Plant Pathology, University of Alicante, Alicante, Spain; ^2^ Centro de Biotecnología y Genómica de Plantas (CBGP, UPM-INIA), Universidad Politécnica de Madrid (UPM)—Instituto Nacional de Investigación y Tecnología Agraria y Alimentaria (INIA), Madrid, Spain

**Keywords:** chitosan, nematophagous fungi, nematode biomanagement, *Pochonia chlamydosporia*, agrobiotechnology, biocontrol

## Abstract

Plants are exposed to large number of threats caused by herbivores and pathogens which cause important losses on crops. Plant pathogens such as nematodes can cause severe damage and losses in food security crops worldwide. Chemical pesticides were extendedly used for nematode management. However, due to their adverse effects on human health and the environment, they are now facing strong limitations by regulatory organisations such as EFSA (European Food Safety Authority). Therefore, there is an urgent need for alternative and efficient control measures, such as biological control agents or bio-based plant protection compounds. In this scenario, chitosan, a non-toxic polymer obtained from seafood waste mainly, is becoming increasingly important. Chitosan is the N-deacetylated form of chitin. Chitosan is effective in the control of plant pests and diseases. It also induces plants defence mechanisms. Chitosan is also compatible with some biocontrol microorganisms mainly entomopathogenic and nematophagous fungi. Some of them are antagonists of nematode pests of plants and animals. The nematophagous biocontrol fungus *Pochonia chlamydosporia* has been widely studied for sustainable management of nematodes affecting economically important crops and for its capability to grow with chitosan as only nutrient source. This fungus infects nematode eggs using hyphal tips and appressoria. *Pochonia chlamydosporia* also colonizes plant roots endophytically, stimulating plant defences by induction of salicylic and jasmonic acid biosynthesis and favours plant growth and development. Therefore, the combined use of chitosan and nematophagous fungi could be a novel strategy for the biological control of nematodes and other root pathogens of food security crops.

## Rhizosphere microbiota and plant health

Ecosystems are largely conditioned by the interaction that plants have with soil-dwelling microorganisms, both beneficial and detrimental (Soliveres and García Palacios, 2019). Approximately 10^8^-10^10^ microbial cells can be found in one gram of soil (Zhang et al., 2017). These complex communities are made up of viruses and various species of archaea, bacteria, and fungi. Soil health depends on biological, as well as physical and chemical factors. The addition of chemical pesticides for extensive agricultural production may compromise the balance between the above factors and damage soil microbial biodiversity (Roesch et al., 2007). Therefore, understanding rhizosphere interactions is essential to design sustainable agricultural strategies respectfully with soil health (Custódio et al., 2022).

**Figure 1:**
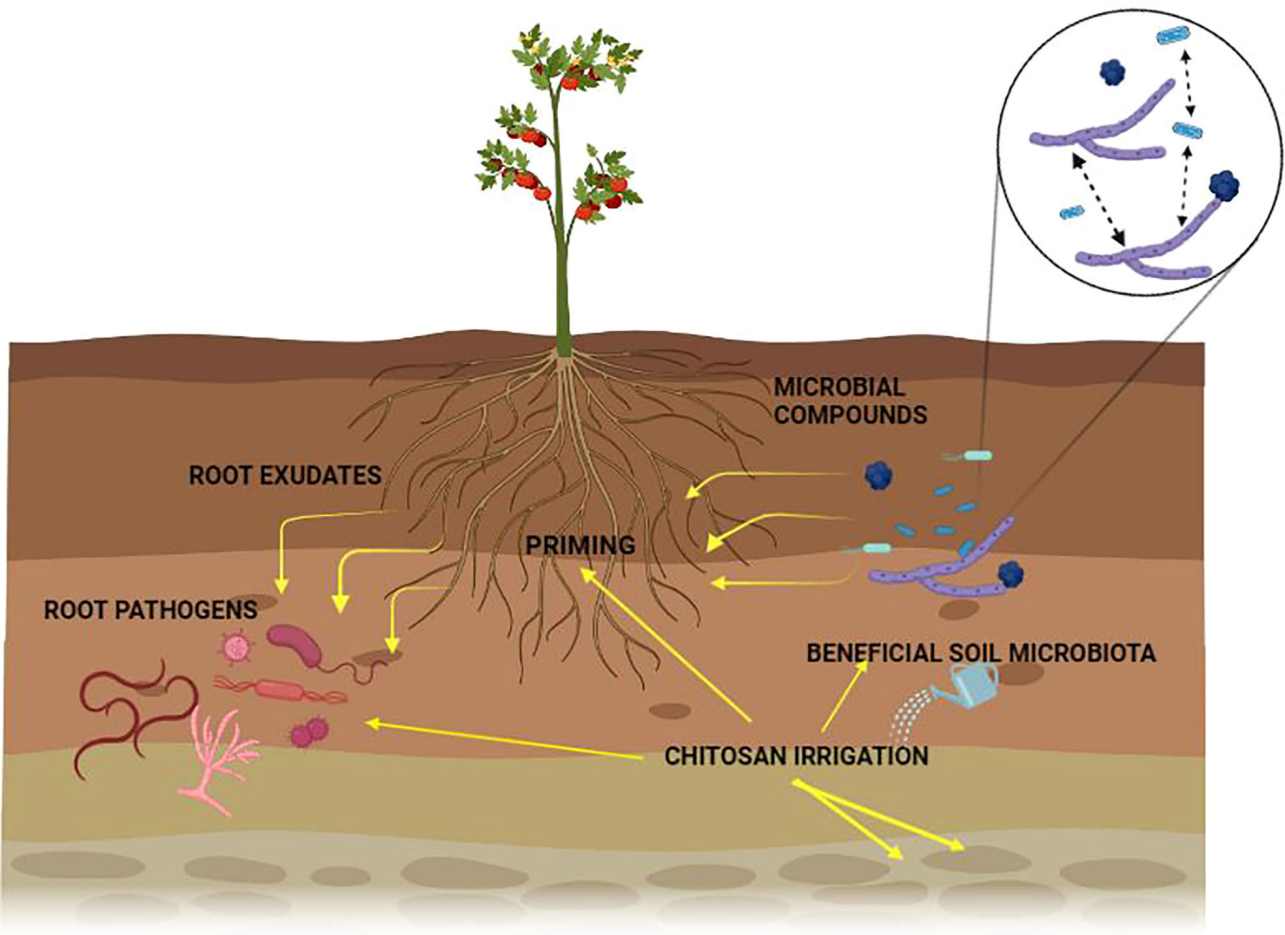
Effect of chitosan on plant-microorganism interactions in the rhizosphere.

**Figure 2:**
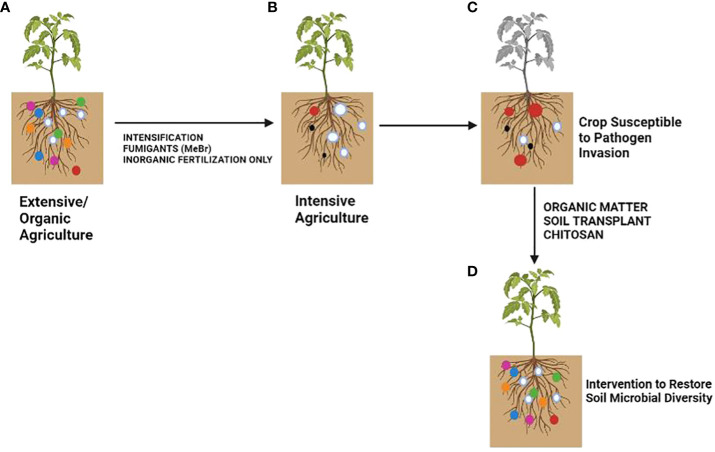
Effect of soil microbiota modification by agricultural practices on crop disease outcome. **(A)** Extensive/Organic farming, **(B)** Intensive farming, **(C)** Crops susceptible to pathogen invasion, **(D)** Soil after microbiota restoration. The colored circles represent different soil microbial taxa. The size represents the amount of inoculum. Pathogenic taxa are indicated by red circles. Modified from Bakker et al. (2012).

Plants rely on other organisms to promote their growth. Beneficial organisms and mutualists include mycorrhizae and endophytic fungi and bacteria (Klein et al., 2007). These organisms increase crop yield (van der Heijden et al., 1998). On the other hand, phytophagous (insects and other herbivores) and pathogens damage plant health (Oerke, 2006). Roots modify the rhizospheric microbiota by secreting organic compounds (mainly secondary metabolites and hormones) in exudates or releasing dead cells (Wardle et al., 2004). Additionally, the soil microbial community can synthesize volatile organic compounds (VOCs) that interact with the multifactorial network of an agroecosystem either to repel pests and diseases or to attract beneficial microorganisms for biological control (**Figure 1**; Lozano-Soria et al., 2020). Crop monocultures may increase disease severity in soils affected by pathogens (Khan and Siddiqui, 2017). Soil microbiota plays a crucial role in the severity of pathogen infections. Thus, if the soil microbial community is not beneficial to a plant, it can increase its susceptibility to disease (**Figure 2**). If a plant grows in association with a rich microbiome over a period co-evolves with it (**Figure 2A**). Under these conditions a complex soil microbiota prevents plant disease (niche exclusion, antibiosis, parasitism/hyperparasitim). Non-specific fumigants may damage soil microbiota and generate poor soils (**Figure 2B**). A poor soil microbiome increases plant susceptibility to pathogen invasion (**Figure 2C**). On the other hand, when soil is treated with organic matter and pest-specific fumigants, the richness of the microbiome is preserved or even increased. This reduces plant susceptibility to pathogen infection even with pathogens present in soil (**Figure 2D**). Therefore, adaptation of the plant to a rich soil microbiome limits pathogen infection (Bakker et al., 2012).

In recent years, agricultural practices have been intensified due to an increased global demand for food, which requires high crop yields. Agricultural intensive practices have a negative impact on soil health, reducing soil fertility and biodiversity, which increases activity of plant pathogens, makes them resistant to chemicals, generates environmental pollution, or soil erosion (Babin et al., 2021).

A healthy soil can keep a proper balance with the environment, sustaining biological productivity and supporting soil micro- and macro-organisms (Larkin, 2015).

Restoring damaged soils is essential to preserve crop quality. Therefore, appropriate agricultural practices must be applied to ensure crop yields for future generations. Establishing an adequate soil microbiota is essential to maintain health of agricultural systems (Sharma et al., 2020). A better mechanistic understanding of rhizosphere interactions can provide new approaches to economically efficient and sustainable plant production (Bakker et al., 2012) while preserving soil biodiversity (Custódio et al., 2022).

## Pathogens threaten global food security

Viruses, Bacteria, Fungi, Nematodes, Protozoa, Algae, and Arthropods can be found in any rhizosphere ecosystem (Mendes et al., 2013). Some of them can cause plant diseases, e.g., root-knot nematodes or cereal take-all caused by the fungus *Gaeumannomyces tritici*. Plants have evolved defence strategies against pest and pathogens. They can, for instance, recognise microbe-associated molecular patterns (MAMPs) and react to them by secreting degradative enzymes or secondary metabolites (Zhou and Zhang, 2020). Plants can also assemble microbial communities to promote pathogen suppression in the rhizosphere (Dudenhöffer et al., 2016). However, these strategies are not always successful. Pathogens have evolved to circumvent plant defences, for instance by secreting effectors (Lo Presti et al., 2015). It has been estimated that *ca.* 40% of global crop production is lost to pests and diseases (FAO, 2021). At least $ 80 billion is lost annually because of plant pathogenic nematodes (Palomares-Rius et al., 2021). Considering the growing population of our Planet (United Nations, 2019) and the reduction of fertile soils due to extensive non-agricultural land use and climate change (Borrelli et al., 2020), avoiding these crop losses due to pests is crucial. Reducing pests and diseases of economically important crops sustainably can help improve the quality of life for farmers, businesses and consumers while respecting the environment.

Plant pathogenic nematodes can cause severe losses in a wide range of crops, e.g., *Meloidogyne* spp. in tomato and banana crops. (Nicol et al., 2011; Silva et al., 2017). These pathogens use living plant resources to grow, compromising crop viability and making nutrient transport difficult leading to plant nutrition deficiency (Abdulkhair and Alghuthaymi, 2016). Chemical nematicides are highly toxic to both the soil and human health (Abawi and Widmer, 2000). Methyl Bromide (MeBr), a soil fumigant, has been used as a nematicide worldwide. MeBr causes ozone layer depletion, so its use has been progressively reduced, and is now completely banned (Nahed et al., 2017). In 1999, the EPA (Environmental Protection Agency) phased out the use of MeBr, except for critical allowable uses agreed in the Montreal Protocol (EPA, 2022). The use of MeBr was revoked in the EU in 2010 (CEE, 2018). Likewise chemical nematicides such as Nemacur^®^ (composed of fenamiphos) and Carbofuran have also been banned for crops. Use of Dichloropropene, a non-specific soil fumigant like MeBr (with or without chloropicrin), has been restricted to fields with no crops (MAPA, 2022). Alternative and effective biological control measures, such as biological control agents or plant protection compounds of biological origin, as well as improved agricultural practices to minimise crop losses are urgently needed as alternatives to chemical control for nematode management (Sikder and Vestergård, 2020).

## Nematode antagonists: biological control

Diverse organisms are natural antagonists of nematodes. For instance, Collembola and Mites predate on plant pathogenic nematodes in soils reducing their populations. In addition, bacteria such as *Pasteuria* spp. can efficiently suppress plant-parasitic nematodes in soils (Stirling, 2018). Nematophagous fungi are highly specialized in nematode parasitism and many of them can be cultured and mass-produced. Our knowledge of these fungi has increased from *ca.* 160 species described at the beginning of the century (Elshafie et al., 2006) to 700 species recently (Soares et al., 2018). Nematophagous fungi infect nematodes by various strategies and have been classified into 5 groups (Soares et al., 2018) accordingly: nematode trappers or predators, egg parasites, endoparasites, toxin-producers and those forming special attack devices (**Table 1**).

**Table 1:** Groups of Nematophagous fungi.

Group	Strategy	Genus Species
Nematode-trapping or Predators	Produce specialised hyphae that trap larviform nematodes. Traps can be adhesive or constricting.	*Arthrobotrys, Dactylellina, Drechslerella, Gamsylella, Hohenbuehelia, Stylopage, Dactylaria, Dactylella, Monacrosporium, Tridentaria, Triposporina, Zoophagus*
Egg-parasites	Use modified hyphal tips (appressoria) for parasitizing nematode eggs, or females. Hydrolytic enzymes, adhesives and fungal metabolites could also be involved.	*Fusarium oxysporum, Purpureocillium* spp., *Paecilomyces* spp.*, Pochonia* spp. (mainly *P. chlamydosporia*), *Metapochonia* spp., *Trichoderma* spp.*, Akantomyces* spp.
Endoparasites	Use spores as infection structures. Spores may attach to the nematode cuticle or be ingested.	*Catenaria anguillulae, Drechmeria coniospora, Esteya* spp., *Haptoglossa* spp., *Hirsutella* spp.
Toxin-producing Fungi	Produce mycotoxins that can paralyze the nematode.	*Climacodon septentrionalis, Hohenbuehelia* spp., *Nematoctonus robustus, Pleurotus* spp.
Producers of Other infection devices	Produce structures to mechanically damage the cuticle of nematodes. A penetration peg is formed to break the cuticle.	*Conocybe lacteal, Coprinus comatus, Stropharia rugosoannulata*

Their parasitic strategies are briefly described. From several sources (Soares et al., 2018; Zhang et al., 2020; Flores Francisco et al., 2021; Li et al., 2021).

Some nematophagous fungi may use more than one strategy for killing nematodes. For instance, *Coprinus comatus* generates “spiny balls” to physically damage the nematode cuticle. Besides, this fungus produces toxins that immobilize and kill nematodes. The combination of both strategies makes nematode attack more efficient (Luo et al., 2007). However, the use of toxin-producing fungi for the management of nematodes in crops has not been widely studied because of possible “non-target” effects (Kumar, 2020). *Pleurotus ostreatus* has recently been shown to induce paralysis of the sensory cilia and calcium accumulation in the nematode, which results in nematode nervous system tissue necrosis (Lee et al., 2005). Brazilian mushrooms (including *Pleurotus* spp.) have been recently found to reduce hatching and mobility of root knot nematode *Meloidogyne javanica* (Hahn et al., 2019). On the other hand, the nematode immune system reacts to the presence of nematophagous fungi (Pujol et al., 2008).

Some nematophagous fungi also enhance plant growth and immunity (Escudero and Lopez-Llorca, 2012), besides their nematode parasitic abilities (Poveda et al., 2020). *Arthrobotrys*, *Aspergillus*, *Catenaria*, *Dactylellina*, *Hirsutella*, *Paecilomyces*, *Pochonia*, *Purpureocillium* and *Trichoderma* (Forghani and Hajihassani, 2020) are reported genera of plant parasitic nematode biocontrol agents. *Pochonia* and *Trichoderma* are good endophytes capable to promote growth, yield, nutrient uptake, and plant development (Zavala-Gonzalez et al., 2015; Mingot-Ureta et al., 2020; Tseng et al., 2020). Both fungi induce plant hormones (jasmonic acid, auxin, and gibberellin) and defence metabolites in tomato, barley, wheat, and *Arabidopsis* (Zavala-Gonzalez et al., 2017; Pentimone et al., 2019; Jaroszuk-Ściseł et al., 2019).

*Trichoderma* spp. are generalist fungi found in soil and roots. They can act as saprophytes (Rashmi et al., 2016), endophytes (Tseng et al., 2020), and pathogens of fungi and nematodes (Sahebani and Hadavi, 2008). This versatility allows them to survive well in the rhizosphere. *Trichoderma* can also induce systemic resistance to nematodes in plants (Medeiros et al., 2017). They are widely used for biocontrol and there are several commercial formulations available worldwide based, mainly, on *T. asperellum*.

*Pochonia chlamydosporia* has also been widely studied as a fungal parasite of eggs and females of plant parasitic nematodes, including cyst and root knot species (*Meloidogyne* spp). *Pochonia chlamydosporia* differentiates appressoria from hyphal tips that adhere to nematode eggshells, allowing penetration and infection (Lopez-Llorca et al., 2002). This process involves proteolytic and chitinolytic activities and secretion of adhesives (Tikhonov et al., 2002). *P. chlamydosporia* has already been used to manage nematodes in food security crops such as tomato (Escudero et al., 2017), potato (Varandas et al., 2020), soybean (Messa et al., 2020), cucumber (Swarnakumari et al., 2020), rice (Khan et al., 2021), banana (Silva et al., 2017) and root beet (Haj Nuaima et al., 2021). *P. chlamydosporia* has also been used for biocontrol of animal parasitic nematodes (Li et al., 2022) and can also infect insects in the laboratory, such as hemipters (Ferraz et al., 2021).

*Pochonia chlamydosporia* also produces compounds suitable for crop protection, such as the insecticide Chlamyphilone (3,4,7-trimethyl-6,8-dioxo-7,8-dihydro-6H-isochromen-7-yl ester; Lacatena et al., 2019) and a wide array of other secondary metabolites with potential applicability (Escudero et al., 2014; Niu, 2017; Suarez-Fernandez et al., 2020). *Pochonia chlamydosporia* is also a root endophyte (Macia-Vicente et al., 2008 *;* Escudero and Lopez-Llorca, 2012; Mingot-Ureta et al., 2020) and can live in the soil as a saprophyte (Bourne et al., 1996). Such versatility allows them to cope with environmental stress (Valadão et al., 2020), which makes it adequate for field application (Sellitto et al., 2016). *Pochonia chlamydosporia* formulations are being developed for practical use to manage plant pests and diseases (Sellitto et al., 2016; Dalla Pasqua et al., 2020; Swarnakumari et al., 2020).

## Chitosan and biological control fungi

Chitosan is a linear polymer of beta-(1-4)-linked N-acetyl-2-amino-2-deoxy-D-glucose and 2-amino-2-deoxy-D-glucose (Kaur and Dhillon, 2014). It is an environmental-friendly biodegradable molecule with interesting features. It is non-toxic to human cells (including lymphocytes; Lopez-Moya et al., 2015) and displays antimicrobial and antioxidant activities (Abd El-Hack et al., 2020). Chitosan has been applied in agriculture (Chakraborty et al., 2020), animal health (Zheng et al., 2020), food (Picciotti et al., 2021), cosmetic (Seino et al., 2020) and medical (Geanaliu-Nicolae and Andronescu, 2020) industries. Chitosan results from the chemical or enzymatic chitin deacetylation. Chitin is present in the cell wall of fungi, in the exoskeleton of arthropods (e.g., insect cuticle) and in nematode eggshells. Chitosan enhances plant growth, development and defence induction under a highly regulated application (Ghasemi Pirbalouti et al., 2017; Lopez-Moya et al., 2017). It also protects plants from infections by pathogenic fungi (Chouhan and Mandal, 2021). This may be due to its plant defence elicitor effect, which can trigger physiological and structural responses in the plant, e.g., substances such as jasmonic acid (JA) and salicylic acid (SA). These mimic the action of plant signalling molecules and produce reactive oxygen species (ROS) that stimulate the plant to produce defence hormones and enzymatic or non-enzymatic antioxidant mechanisms to mitigate ROS damage to cells (Lopez-Moya et al., 2015; Lopez-Moya et al., 2019; Suarez-Fernandez et al., 2020). To this respect, induction of plant defences by chitosan also reduces severity of root-knot nematodes (*Meloidogyne*) in plants (Vasyukova et al., 2003, Escudero et al., 2017).

*Trichoderma* spp. and *P. chlamydosporia* genomes encode, hydrolases such as, proteases, chitosanases and chitinases (Kappel et al., 2020). *Pochonia chlamydosporia* genome encodes the largest set of chitosanases found in fungi (Larriba et al., 2014; Aranda-Martinez et al., 2016). Their use in tandem with chitin deacetylases could explain the ability of the fungus to degrade nematode eggshells. (Lopez-Moya et al., 2019; Lopez-Llorca et al., 2020). Combinations of chitosan and biocontrol (entomopathogenic and nematophagous) fungi are currently developed for enhancing fungal pathogenicity and plant defence abilities. This has been reported for *Beauveria bassiana* (Abdullah and Sukar, 2021) and *Metarhizium anisopliae* (Wu et al., 2021). Nematophagous fungi have also demonstrated to be more efficient for biological control when combined with chitosan than on their own (Escudero et al., 2017; Singh and Chittenden, 2021; Kappel et al., 2022). For *P. chlamydosporia*, addition of chitosan increases nematode parasitism by induction of appressoria differentiation on nematode eggshell (Escudero et al., 2016 and Escudero et al., 2017).

### Chitosan: Mode of action in plants and fungi

**Figure 3:**
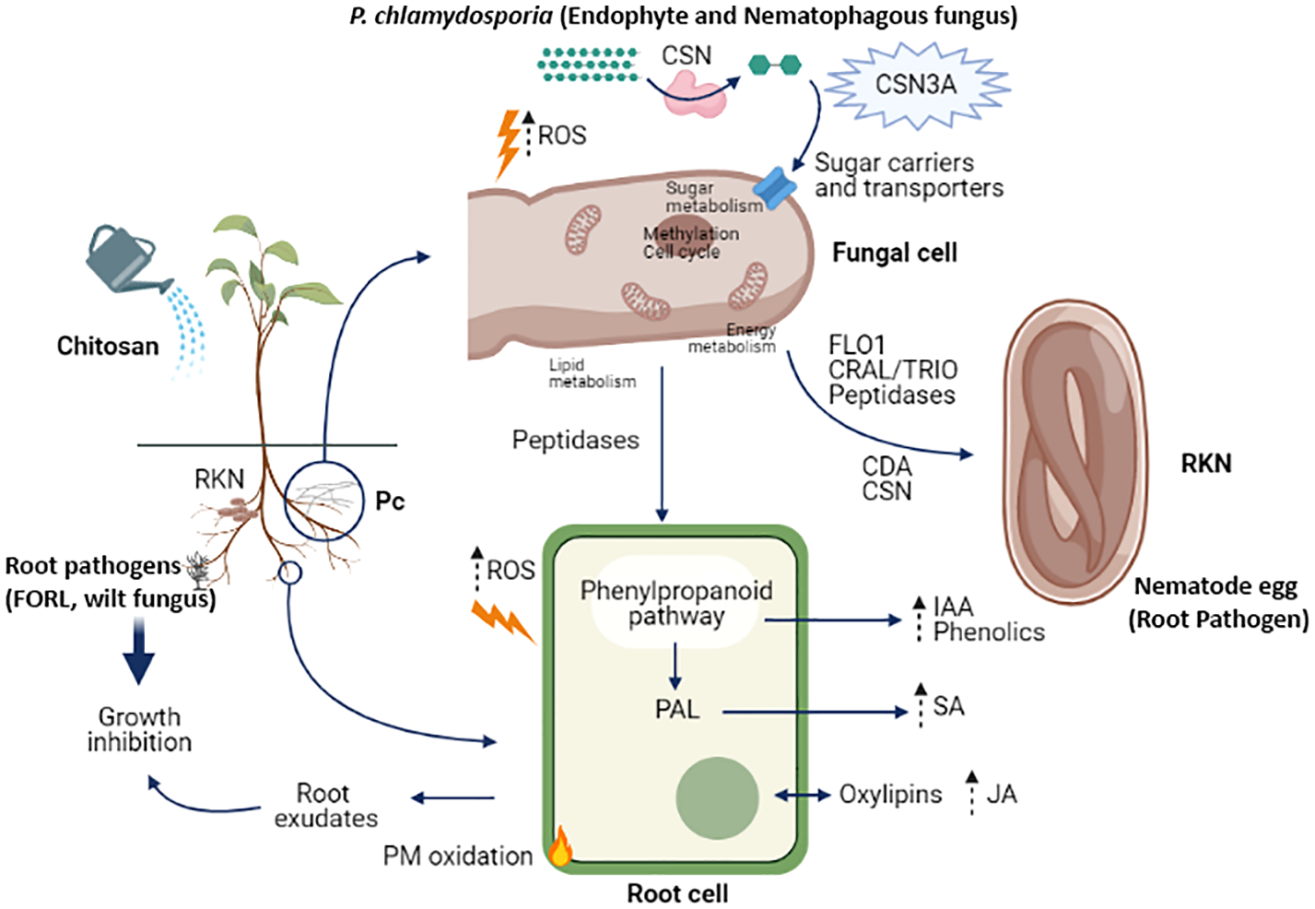
Cell and molecular effects of chitosan in the rhizosphere. Abbreviations: RKN (root-knot nematodes), FORL (*Fusarium oxysporum* f. sp. radicis lycopersici), ROS (reactive oxygen species), CSN (chitosanase), CSN3A (chitosanase 3 isoform A), CDA (chitin deacetylase), IAA (Indole-3-acetic acid), SA (salicylic acid), JA (jasmonic acid), PAL (phenyl ammonia lyase), PM (plasma membrane).

Roots in the simulation (**Figure 3**) are colonized by a biocontrol fungus (*P. chlamydosporia;* Pc) and infected by a wilt pathogenic fungus (*Fusarium oxysporum* f. sp. radicis-lycopersici; FORL) and root knot nematodes (RKN). Chitosan depolarises the plasma membrane of plant root cells and increases reactive oxygen species (ROS). This is linked with an increase in jasmonic acid secretion and other oxylipins in root exudates (Suarez-Fernandez et al., 2020). Furthermore, chitosan permeabilises the plasma membrane of susceptible fungi (Palma-Guerrero et al., 2010; Hernández-Lauzardo et al., 2011) by generating reactive oxygen species (ROS) which resulted toxic to the organism (Lopez-Moya et al., 2015). This would activate the phenylpropanoid acid pathway, increasing the secretion and accumulation of phenolic compounds (Xoca-Orozco et al., 2019) and plant hormones (Kwak et al., 2006), such as indoleacetic acid. Evidence for activation of the phenyl ammonia lyase (PAL) pathway is consistent with the increase in salicylic acid found in root exudates in chitosan treated plants. These hormones could also accumulate in root tissues (Lopez-Moya et al., 2017). Root exudates of chitosan-treated plants inhibit root pathogens, such as FORL or nematodes (Suarez-Fernandez et al., 2020). The nematophagous fungus *P. chlamydosporia* in the rhizosphere also generates ROS in response to chitosan. *P. chlamydosporia* alters plant metabolite secretion to establish endophytic interactions enhancing plant defences (de Souza Gouveia et al., 2022). The fungal cell secretes chitosanases to degrade chitosan and increases the synthesis of sugar transporters to assimilate the catabolites of chitosan depolymerisation (Suarez-Fernandez et al., 2021). Alternative splicing events occur in *P. chlamydosporia* chitosanase encoding genes (e.g., *csn3*), probably to increase the efficiency of chitosan degradation (Sambles et al., 2022). When nematode eggs are present in the system, chitosan induces genes and proteins involved in adhesion, eggshell degradation, and parasitism (Escudero et al., 2016; Suarez-Fernandez et al., 2021). Increased secretion of peptidases or LysM effectors from the fungus could also be related to increased endophytism of the fungus in the plant root, favouring this fungal lifestyle and effective protection against nematodes and other plant pathogens. To summarize, the elicitor effect of chitosan on plant defences, as well as the enhancement of proteases and adhesives in *P. chlamydosporia* nematode egg infection, support the combined use of biocontrol fungi and chitosan for plant protection to important pests and diseases.

## Application of chitosan nanoemulsions and nematophagous fungi for nematode management

Natural plant protection compounds with low environmental toxicity have a clear niche in agrobiotechnology. Some biocontrol agents such as *P. chlamydosporia* or *B. bassiana* are enhanced by chitosan which also acts as a plant defence elicitor. Due to the ban on many chemical plant protection compounds, chitosan-derived products started to be used in 1990 against plant diseases in crops and during post-harvest (Yin and Du, 2011). Recently, the European Union has recognised chitosan as an active compound suitable for agricultural use. Most plant protection products are hydrophobic, so their formulation requires the use of chemicals to keep their physicochemical properties (Liang et al., 2018). Nanoemulsions are small droplets dispersed in an immiscible fluid using a suitable emulsifier. They have recently generated interest in agrochemical fields due to the improved bioavailability of poorly water-soluble pesticides (Chadha, 2021). Zn-Chitosan nanoparticles (microscopic particles encapsulating a compound), reduce the severity of infection of *Curvularia lunata* infecting maize leaves, and Zn-chitosan also enhances the growth of nanoparticulate maize seedlings (Saharan et al., 2016; Choudhary et al., 2019).

Chitosan nanoparticles can also be used as coating agents for post-harvest disease prevention. Ripening bananas coated with 0.2% chitosan nanoparticles show a slower skin discolouration than untreated controls (Esyanti et al., 2019).

*P. chlamydosporia* can reduce damage caused by *M. incognita* on tomato plants in a single application (Yang et al., 2012). Chitosan has been combined with *P. chlamydosporia* for root knot nematode management in tomato (Escudero et al., 2017). Plants treated with chitosan had fewer galls and reduced nematode multiplication compared to controls. *Pochonia chlamydosporia* was the only fungus identified parasitizing nematode eggs in chitosan-treated soils. Soils in fields with root knot nematodes and *P. chlamydosporia* irrigated with chitosan showed 36.6% egg parasitism compared to 29.3% in non-irrigated and non-sterilised soils (Escudero et al., 2017). Soils in which *P. chlamydosporia* is naturally present treated with chitosan are expected to show increased colonisation of plant roots and increased parasitism of eggs by the fungus. This strategy could sustainably reduce the population of plant-parasitic nematodes in agroecosystems.

Our challenge would be to formulate chitosan and *P. chlamydosporia* in a suitable way for creating *de novo* nematode suppression in agroecosystems. Such studies are underway in our laboratory.

The application of microparticles containing chemicals and biological agents is an innovative approach for plant pests and diseases management (Jurić et al., 2021). Encapsulation of biocontrol agents in chitosan microparticles ensures their protection, survival, and selective delivery for pest management. Combining liquid or particulate chitosan formulations with the nematophagous fungus *P. chlamydosporia* is an effective way to recycle waste from seafood industries while generating a sustainable way for managing nematode infections in the field.

## Conclusions and future prospects

Nematophagous fungi in combination with chitosan represents a new solution for sustainable management of plants parasitic nematodes which cause significant economic losses in crops worldwide. Understanding molecular interactions between organisms in the rhizosphere is essential to determine the best approach to manage crop pathogens without harming agroecosystems. The combination of chitosan with nematophagous fungi still needs to be explored further to determine the best formulation and timing for field applications, but it constitutes a promising tool for the sustainable management of pests and diseases of food security crops.

## Author contributions

RL-N and MS-F conducted the bibliographic search, organised, and summarised the information, and wrote the manuscript draft. Supervision, methodology, co-writing (original draft), and reviewing: FL-M. Supervision, methodology, co-writing (original draft), reviewing and funding acquisition: LL-L. All authors have read and agreed to the published version of the manuscript.

## Funding

This research was funded by PID2020-119734RB-I00 Project from the Spanish Ministry of Science and Innovation and by European Project H2020 MUSA no. 727624.

## Acknowledgments

We would like to thank the Plant Pathology Laboratory members of the University of Alicante for their help and support.

## Conflict of interest

The authors declare that the research was conducted in the absence of any commercial or financial relationships that could be construed as a potential conflict of interest.

## Publisher’s note

All claims expressed in this article are solely those of the authors and do not necessarily represent those of their affiliated organizations, or those of the publisher, the editors and the reviewers. Any product that may be evaluated in this article, or claim that may be made by its manufacturer, is not guaranteed or endorsed by the publisher.
